# T-helper 1-type cytokines induce apoptosis and loss of HER-family oncodriver expression in murine and human breast cancer cells

**DOI:** 10.18632/oncotarget.10298

**Published:** 2019-10-15

**Authors:** Prachi Namjoshi, Lori Showalter, Brian J. Czerniecki, Gary K. Koski

**Affiliations:** ^1^Department of Biological Sciences, Kent State University, Kent, Ohio, USA; ^2^Department of Surgery, University of Pennsylvania, Philadelphia, Pennsylvania, USA

**Keywords:** apoptosis, HER-2, T-helper 1, IFN-γ, TNF-α

## Abstract

A recent neoadjuvant vaccine trial for early breast cancer induced strong Th1 immunity against the HER-2 oncodriver, complete pathologic responses in 18% of subjects, and for many individuals, dramatically reduced HER-2 expression on residual disease. To explain these observations, we investigated actions of Th1 cytokines (TNF-α and IFN-γ) on murine and human breast cancer cell lines that varied in the surface expression of HER-family receptor tyrosine kinases. Breast cancer lines were broadly sensitive to the combination of IFN-γ and TNF-α, as evidenced by lower metabolic activity, lower proliferation, and enhanced apoptosis, and in some cases a reversible inhibition of surface expression of HER proteins. Apoptosis was accompanied by caspase-3 activation. Furthermore, the pharmacologic caspase-3 activator PAC-1 mimicked both the killing effects and HER-2-suppressive activities of Th1 cytokines, while a caspase 3/7 inhibitor could prevent cytokine-induced HER-2 loss. These studies demonstrate that many **in vivo** effects of vaccination (apparent tumor cell death and loss of HER-2 expression) could be replicated **in vitro** using only the principle Th1 cytokines. These results are consistent with the notion that IFN-γ and TNF-α work in concert to mediate many biological effects of therapeutic vaccination through the induction of a caspase 3-associated cellular death mechanism.

## Introduction

The human epidermal growth factor receptor family is comprised of four known members (HER-1-4). They each perform a variety of normal physiological functions, but can also serve as oncodrivers in tumorigenesis [1, 2]. Of these, HER-2 and HER-3 are particularly inter-dependent proteins, since by themselves they are functionally incomplete receptors. For example, HER-2 has no known ligand, while HER-3 lacks kinase activity; but as a heterodimer, they form a highly efficient functional unit that constitutes the most active signaling dimer in this family [3]. HER-2 overexpression in breast cancers is associated with invasiveness, poor prognosis and resistance to chemotherapy [4, 5]. HER-3 also plays a critical role in tumor cell growth and proliferation in tumors driven by HER-2 over-expression [6, 7]. HER-2 and HER-3 are therefore attractive targets for novel breast cancer treatments, both pharmacological and immunological.

A previous DC1-polarized dendritic cell-based neoadjuvant vaccine trial to treat early breast cancer (HER-2^pos^ ductal carcinoma *in situ*; DCIS) generated strong and durable Th1-polarized immunity against HER-2 [8, 9]. Of 27 evaluable patients, five had complete responses (i.e. no evidence of tumor at the time of surgery). In addition, within the patient subset with residual DCIS at the time of surgery, there was evidence of reductions in the area of disease for a number of individuals [10]. Another interesting observation was that for about half of these remaining patients, levels of HER-2 expression decreased, often to nearly undetectable levels [8, 10]. These alterations in the tumors were accompanied by lymphocytic infiltrations into the diseased areas of the breast. Of T lymphocytes, CD4^pos^ Th cells made up the majority of infiltrating cells; CD8^pos^ CTLs on the other hand were relatively few. Substantial B-cell infiltrates were also observed. Taken together, these observations suggest an immune-mediated destruction and alteration of tumor cells in many of the vaccinated subjects. At the time of these studies, we interpreted the observed losses of HER-2 expression to be a special vaccine-induced form of the “immunoediting” phenomenon described previously by others [11, 12]; HER-2-specific CTL were presumably destroying the HER-2-overexpressing cell population within heterogeneous tumor masses and thus selecting for a residuum of disease that was HER-2^low/neg^ and thus poor targets for continued destruction by the CTL. Although this appeared the most reasonable explanation at the time, there were several unsatisfying elements in this narrative. For example, this reasoning required tumor cell death to be mediated by MHC class I-restricted CTL, yet the CD8^pos^ infiltrates seemed somewhat sparse to account for all the changes to the tumors. In contrast, there were quite sizable infiltrates of CD4^pos^ cells. However these Th cells should not be able to recognize tumor cells directly, due to their MHC Class II-restricted nature, and besides most of the CD4^pos^ cells were observed to congregate just outside the diseased ducts [10] rather than intermingling with the tumor.

An alternate explanation would be that the observed infiltrating MHC class II-expressing B cells would present shed tumor antigens to the vaccine-induced anti-HER-2 CD4^pos^ Th1 cells, and these in turn would produce a soluble factor(s) that could diffuse into the tumor bed and mediate the observed biological effects on the tumor cells. This would allow the Th cells to affect the tumor without direct contact and recognition. But if secreted factors were involved, which ones could they be? The principle Th1 cytokines are IFN-γ and TNF-α, and there is considerable evidence that these can have effects on tumor cells [13, 14]. Indeed, a recent manuscript showed that the paired combination of IFN-γ and TNF-α could induce a state of permanent growth arrest in some murine and human cancer cells consistent with senescence [15]. We therefore hypothesized that the combination of IFN-γ and TNF-α could replicate, *in vitro*, the observed anti-breast tumor effects of DC1 vaccination, including induced cell death with associated loss of HER-2 expression. We show in the present studies that the combination of these cytokines can indeed induce in a number of HER family-expressing murine and human breast cancer lines apoptosis, as well as a strong suppression of HER expression, both of which are associated with the activation of caspase-3. The demonstrated *in vitro* action of these paired cytokines can therefore account for most of the observed changes that occur in HER-2^pos^ DCIS as a consequence of Th1 immunity induced through polarized DC1 vaccination.

## Results

### Th1 cytokines prevent growth of murine breast cancer lines

**Figure 1:**
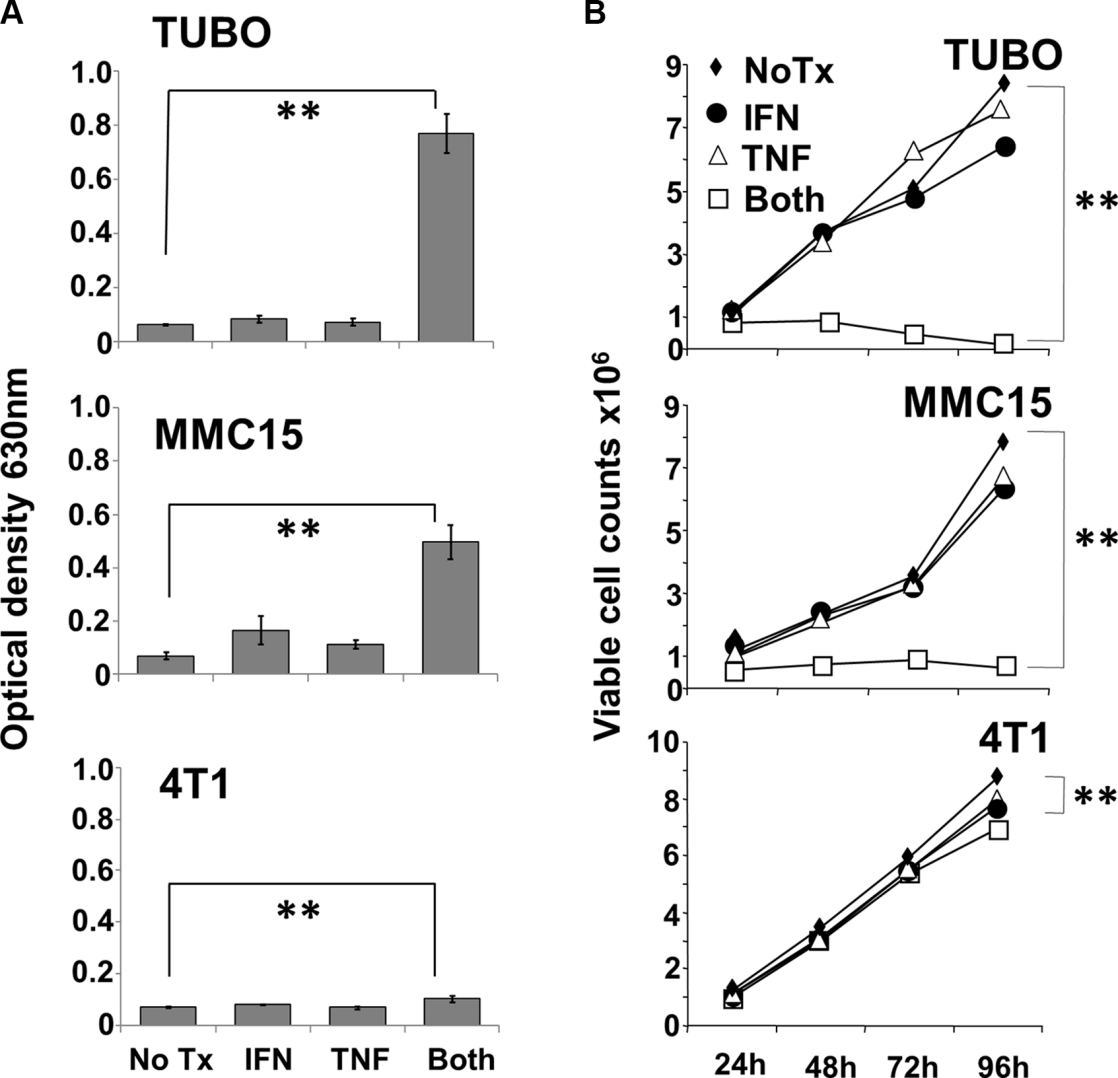
Effect of Th1 cytokines on metabolic activity and proliferation of murine breast cancer cell lines. TUBO (rHER-2^pos^), MMC15 (rHER-2^pos^) and 4T1 (rHER-2^neg^) cell lines were cultured for 96 hours in the presence of IFN-γ (12.5 ng/ml), TNF-α (1 ng/ml), both cytokines, or left untreated. (**A**) at the 96 hour point, 20 μl Alamar Blue dye (resazurin; 0.7 mg/ml stock solution) was added for 6 additional hours, after which optical densities of supernatants were read at 630 nm (^**^ *p* </=.01).Results from 3 independent experiments +/− SEM. (**B**) Replicate wells were harvested after 24, 48, 72 and 96 hours of culture, and stained with Trypan Blue. Dye-excluding cells were enumerated microscopically with the aid of a hemocytometer (^**^ *p* </=.01). Results from 3 independent experiments +/− SEM.

To study the effect of TNF-α and IFN-γ on murine rHER-2^pos^ breast cancer cells, TUBO and MMC15 lines were cultured in the presence of either or both cytokines for up to 96 hours. The rHER-2^neg^ 4T1 line was likewise tested for comparison. Initial studies assessed cell response to cytokines via the Alamar Blue assay, which measures metabolic activity of cells through reduction of the Alamar Blue dye, a change that can be followed spectrophotometrically. We found that both TUBO and MMC15 cell lines metabolized the alamar blue dye at comparable levels when left untreated, or treated with single cytokines (Figure 1A upper and middle panels). However, when treated with both IFN-γ and TNF-α, metabolic activity was dramatically suppressed (*p* < .001). For 4T1 cells, the differences in metabolic activity between untreated and dual cytokine treated cells were still statistically significant (*p* < .01), yet very small in magnitude, indicating a relative insensitivity to the cytokines (Figure 1A lower panel).

The Alamar Blue assay indicates differences in metabolic activity between treatment groups, but cannot distinguish whether contrasts are due to variances in aerobic respiration between groups of equally viable cells, differences in cell proliferation between groups over the culture interval, or differences caused by actual cell death. We therefore sought to gain information on cell viability through vital staining and direct microscopic observation of cultures. Cultured cells were treated as before, and observations made every 24 hours over the course of four days. Cultured cells were either harvested each day for Trypan Blue staining and counting, or were subjected to direct photomicroscopy *in situ*. Trypan blue staining of TUBO and MMC15 cells revealed that for untreated or single cytokine-treated groups, cell counts increased steadily over the course of four days (Figure 1B upper and middle panels). For dual cytokine-treated groups, however, no increases in number were seen for either MMC15 or TUBO cells over the course of 96 h. In contrast, 4T1 cells continued to grow vigorously under all conditions (Figure 1B lower panel), and although only small distinctions were apparent at 96 hours between untreated and dual cytokine-treated cells, the difference was nonetheless statistically significant (*p* < .01). Nonetheless, a dramatic contrast in sensitivity to Th1 cytokines was apparent for 4T1 cells compared with TUBO or MMC15 cells.

**Figure 2:**
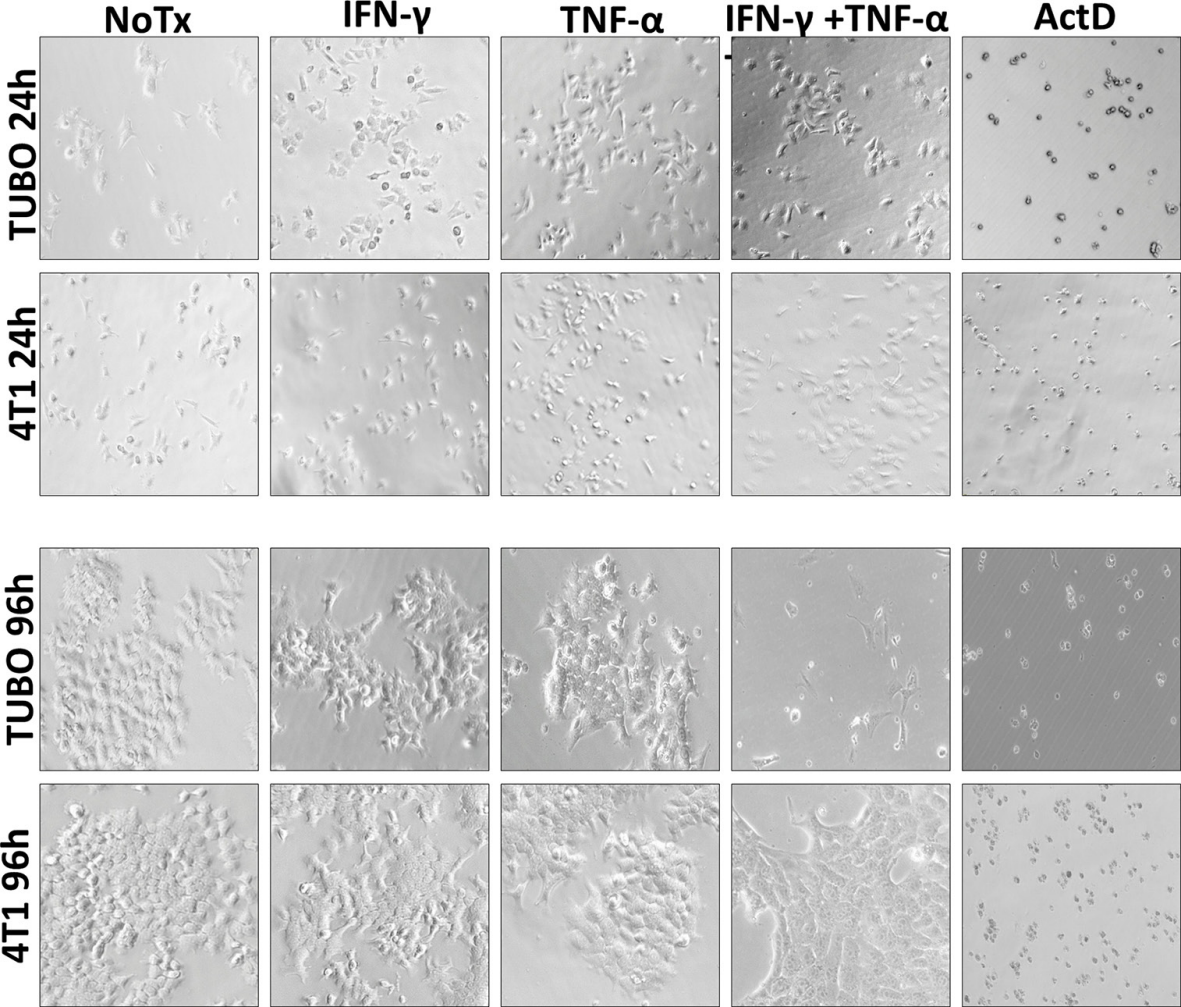
Photo microscopic study of Th1 cytokine-treated murine breast cancer cells. TUBO (HER-2^pos^) and 4T1 (HER-2^neg^) cell lines were cultured for in the presence of IFN-γ (12.5 ng/ml), TNF-α (1 ng/ml), both cytokines, or left untreated. Cells were subjected to photomicroscopy at 24 and 96 hours of culture.

Microscopic examination of cultured cells told a similar story. At 24 hours, little difference could be distinguished between the four treatment groups for either TUBO or 4T1 cells (Figure 2 upper panels). Even at 48 and 72 hours, although cells were clearly not multiplying, only modest evidence of cell death was apparent by visual inspection (data not shown). However, by 96 hours, not only did TUBO cells treated with both IFN-γ and TNF-α show few adherent, viable cells, but many dead cells were also apparent (Figure 2 lower panels). Similar observations were made for MMC15 cells (data not shown). In contrast, no differences could be discerned between any treatment groups for 4T1 cells, confirming a relative lack of sensitivity to Th1 cytokines.

### Differences in sensitivity to Th1 cytokines do not result from differential expression of cytokine receptors

We next wanted to rule out the possibility that the differences in sensitivity between TUBO and 4T1 lines could be explained simply by differential expression of Th1 cytokine receptors. We therefore stained both lines with fluorescently-labeled antibodies against IFN-γR1, TNF-αR1, or an isotype-matched control antibody, and analyzed the cells via flow cytometry (Supplementary Figure 1). We found that both cell lines displayed modest, yet comparable levels of cytokine receptors, indicating that the differences in sensitivity between the two lines cannot be explained by differential expression of cytokine receptors.

### *Th1 cytokines induce* apoptotic cell death

**Figure 3:**
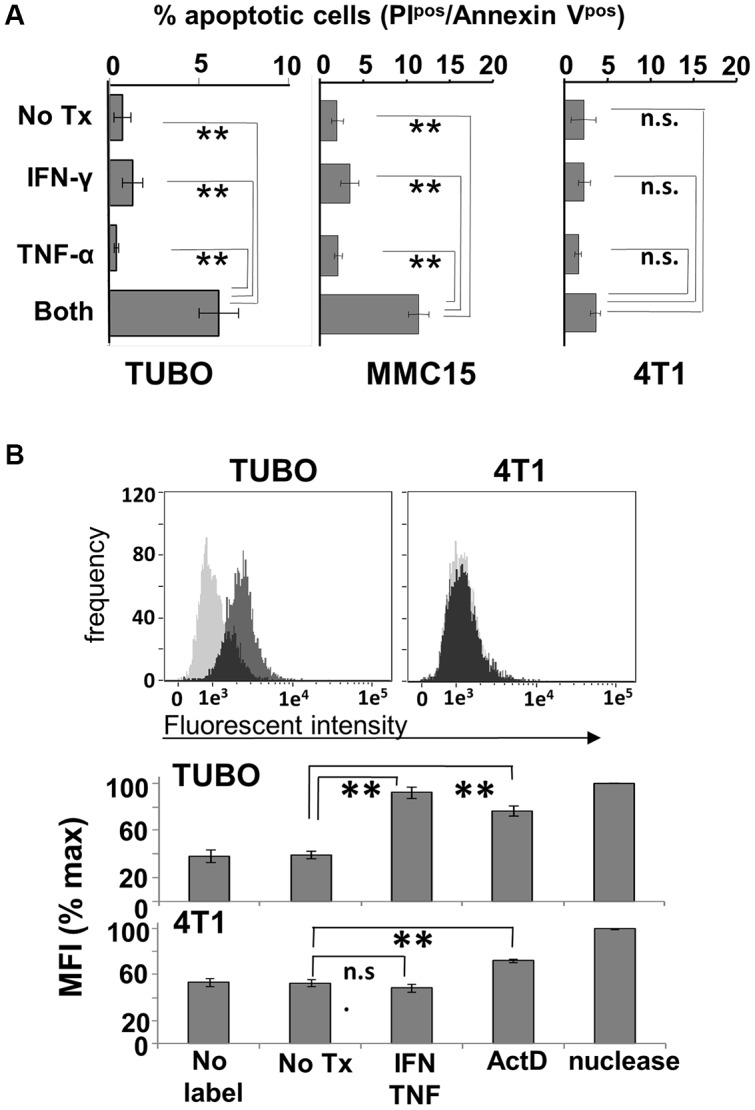
Induction of apoptosis by Th1 cytokines. (**A**) TUBO, MMC15 and 4T1 cells left untreated, or treated with TNF-α (1 ng/ml), IFN-γ (12.5 ng/ml) or both cytokines and cultured for 96 hours. Cells were then harvested and stained with Annexin V and PI and subjected to flow cytometric analysis. Values represent percentage of double-staining (apoptotic) cells +/− SEM. (**B**) TUBO and 4T1 cells were cytokine-treated and cultured as before. Harvested cells were formaldehyde-fixed and labeled with biotinylated nucleotides, then stained with FITC-labeled streptavidin and subjected to flow cytometric analysis. Upper panels display histogram analysis from a single representative of labeling for untreated (gray trace) versus cytokine-treated (black trace) cells. Lower panel represents summary analysis of 3 separate experiments, expressed as percent maximum mean fluorescent index +/− SEM (^**^ *p* </=.01; n.s. not significant).

To determine whether the effects of Th1 cytokines are due to induction of apoptosis, TUBO, MMC15 and 4T1 cells were once again cultured with no treatment, or exposed to single or dual Th1 cytokines. Cells were then harvested at 72 hours post-treatment and stained with FITC-AnnexinV and propidium iodide (PI), then subjected to flow cytometric analysis. These studies showed that TUBO and MMC15 cells treated with both IFN-γ and TNF-α displayed significantly greater populations of AnnexinV^pos^/PI^pos^ (apoptotic) phenotype, as compared with untreated cells or single cytokine-treated cells (Figure 3A). On the other hand, 4T1 cells did not display significantly enhanced levels of AnnexinV^pos^/PI^pos^ cells in response to Th1 cytokines, indicating insensitivity to cytokine-induced apoptosis.

We confirmed the apoptotic nature of cytokine-induced cell death via the TUNEL assay, which detects DNA damage through enzyme-mediated repair with a biotin-labeled nucleotide analog, the incorporation of which can be detected using fluorescently-labeled streptavidin via flow cytometry. Here, TUBO and 4T1 cells were left untreated, or treated with dual Th1 cytokines for 96 hours, harvested, subjected to TUNEL labeling, and analyzed by flow cytometry (Figure 3B, upper panels). It was evident via histogram analysis that the incorporation of labeled nucleotide was increased for cytokine-treated TUBO cells (dark histogram traces) compared with untreated cells (light histogram traces). In contrast, for 4T1 cells, the fluorescent intensity for untreated and cytokine-treated cells were virtually identical, as evidenced by the overlapping nature of their respective histograms, indicating no differences in labeled nucleotide incorporation. Analysis of 3 independent experiments including nuclease- and Actinomycin D-treated positive controls showed that statistically significant (*p* < .0001) enhancements in labeling occurred only with cytokine-treated TUBO cells but not 4T1 cells (Figure 3B, lower panels), even though both lines showed enhanced incorporation of label after treatment with Actinomycin D. These studies provided strong evidence that Th1 cytokine-induced death was occurring through an apoptotic mechanism.

### Th1 cytokines induce caspase-3 activation

**Figure 4:**
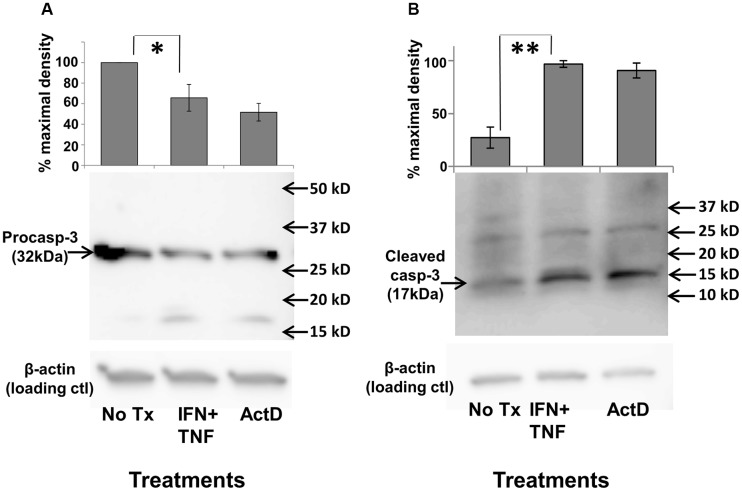
Th1 cytokine-induced activation of caspase-3. TUBO cells were cultured alone, in the presence of TNF-α plus IFN-γ, or with actinomycin D for 5 hours, harvested, and subjected to Western blot analysis using anti-procaspase 3 (**A**), or anti-activated (cleaved) caspase-3 (**B**). Upper panels represent combined data from 3 separate experiments +/− SEM; lower panels contain sample Western blot from a single representative experiment (^*^ *p* </= .05; ^**^ *p* </=.01).

To determine critical components of the underlying pathway for cellular apoptosis, we investigated the involvement of executioner caspases, which are intimately linked to most known downstream apoptotic processes. Caspases exist in inactive, high molecular weight pro-forms which must be cleaved proteolytically into active, lower molecular weight, components. Both of these forms can be individually detected via Western Blot analysis. Cultured TUBO and 4T1 cells were treated with either dual Th1 cytokines, actinomycin D (positive control) or left untreated. After 5 hours, cells were harvested, extracted, proteins separated via SDS-PAGE and analyzed via Western Blot for expression of pro-caspase 3 (32kDa form), activated caspase3(17kDa form) and β-actin (loading control). For TUBO cells, we found that dual Th1 cytokine treatment resulted in statistically-significant (*p* < .03) decrease in pro-caspase 3 levels, comparable to that induced by actinomycin D (Figure 4A), while levels of the active form correspondingly and significantly (*p* < .001) increased (Figure 4B). In contrast, we did not detect any cytokine-induced diminution of procaspase 3 in the insensitive 4T1 cells (Supplementary Figure 2), nor did we detect activation of other caspases, including caspase 1, caspase 6 and caspase 7 in TUBO cells (data not shown). These studies show that treatments that induce apoptosis in sensitive lines also activate the executioner caspase, caspase-3, thereby suggesting that it may have a role in apoptotic cell death induced by Th1 cytokines.

### Th1 cytokines induce down-regulation of surface HER receptors for many breast cancer lines

**Figure 5:**
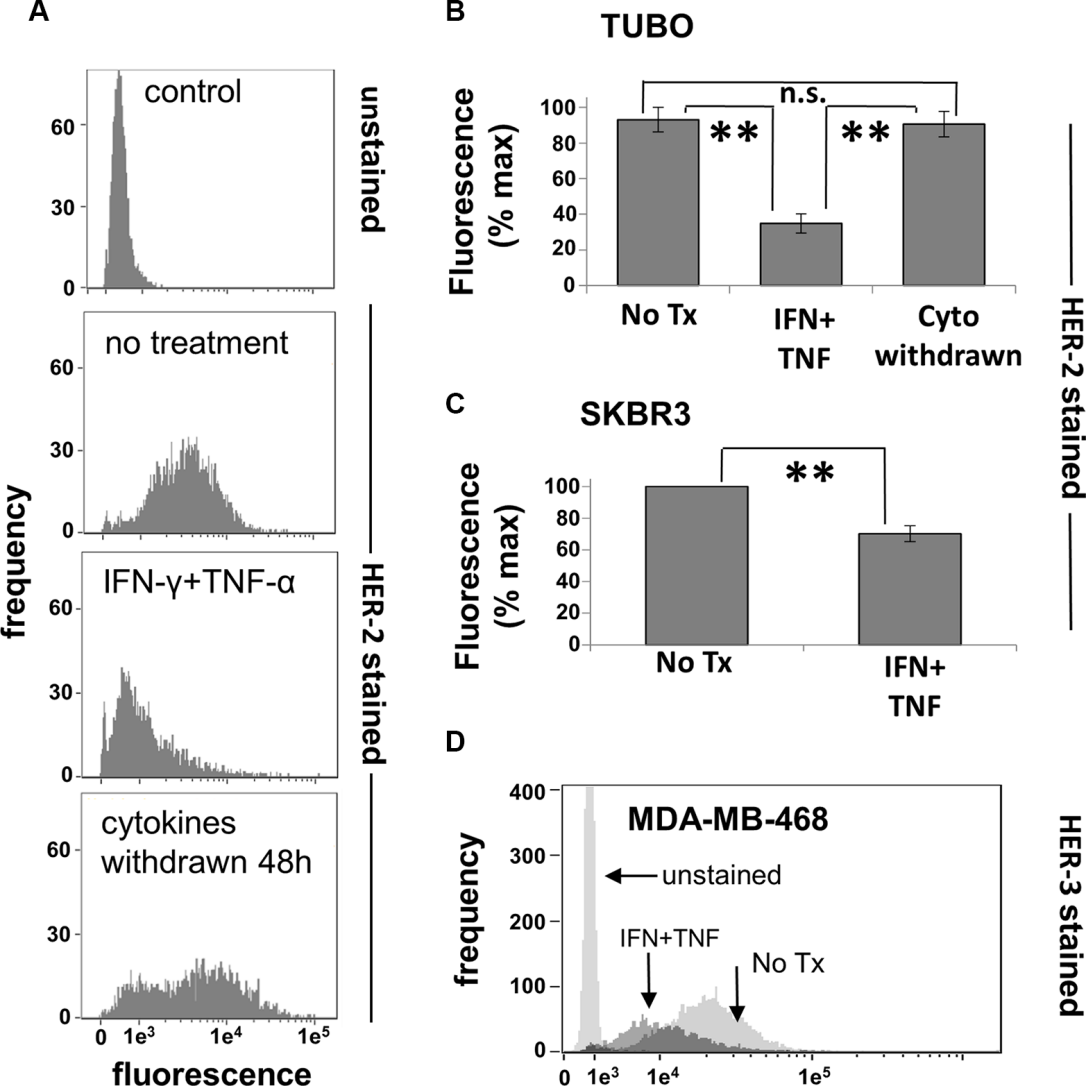
Th1 cytokines alter HER-family expression on murine and human breast cancer cells. (**A**)TUBO cells were cultured alone or in the presence of TNF-α and IFN-γ for 72 hours, harvested and analyzed for HER-2 expression via flow cytometry (upper 3 panels). Replicate treated wells were washed free of cytokines at the 72 hour point and cultured an additional 48 hours, demonstrating the recovery of HER-2 expression (lower panel). (**B**) Summary of 3 separate trials with TUBO cells illustrating cytokine-induced HER-2 loss as well has recovery after cytokine withdrawal. Values represent percent maximal fluorescence +/− SEM from 3 separate experiments. (**C**) Human HER-2^pos^ SKBR3 cells were cultured alone or with TNF-α (1 ng/ml) plus IFN-γ (12.5 ng/ml) for 72 hours, harvested, and analyzed for HER-2 expression via flow cytometry. Values represent percent maximal fluorescence +/− SEMfrom 3 separate experiments. (**D**) Human HER-2^neg^/HER-3^pos^ MDA-MB-468 breast cancer cells were cultured alone or in the presence of TNF-α plus IFN-γ for 72 hours, harvested, and analyzed for HER-3 expression via flow cytometry (^**^ *p* </=.01).

We demonstrated in the previous experiments that the combination of IFN-γ and TNF-α was capable of inducing apoptotic cell death in rHER-2^pos^ cell lines, confirming the first part of our hypothesis that soluble factors secreted by Th1 cells could account for the clinical effects of DC1 vaccination. We now turned our attention to the effect of these cytokines on the expression of HER family members. We began our studies with the TUBO line. Cultured cells were treated with cytokines, incubated for 72 hours (a time point preceding maximal cell death), harvested, and stained with anti-rHER-2 antibodies. Flow cytometric analysis showed that dual cytokine treatment led to a strong down-modulation of surface rHER-2 expression in these cells; a representative experiment is shown in Figure 5A. This loss was selective for rHER-2, since levels of the common epithelial cell marker EpCAM were also monitored and not only failed to drop, but actually increased slightly (Supplementary Figure 3). Interestingly, when cytokines were removed after this 72-hour exposure (prior to widespread cell death) by replacing the culture medium, the surviving rHER-2 suppressed cells were able to recover over the course of 48 hours of additional culture to begin proliferating and re-expressing surface HER-2 (Figure 5A lower panel). This experiment was repeated a total of 3 times, with statistically significant HER-2 loss induced by dual cytokine treatment (*p* < 0.0001), and recoveries of rHER-2 expression after cytokine withdrawal not significantly different (*p* = .443) from untreated cells (Figure 5B). We also examined human breast cancer cell lines for cytokine-induced suppression of surface HER family members. The HER-2^pos^ line SKBR3 demonstrated somewhat less dramatic, yet statistically-significant reductions (*p* < 0.005) in HER-2 surface expression (Figure 5C), while the HER-2^neg^/HER-3^pos^ cell line MDA-MB-468 showed strong down-regulation of surface HER-3 expression (Figure 5D) in response to paired Th1 cytokines. It should be noted that not all breast cancer cell lines tested posted significant losses in HER-family expression. For example, murine MMC15 cells retained baseline HER-2 expression despite sensitivity to cytokine-induced cell death (not shown). These studies nonetheless indicate that the combination of IFN-γ and TNF-α are capable of inducing, for many breast cancer lines, reductions in surface expression of HER family proteins *in vitro*, similar to what is observed *in vivo* with DC-based vaccinations that induce strong Th1 immunity.

### Loss of HER-2 expression is associated with apoptotic cell death

**Figure 6:**
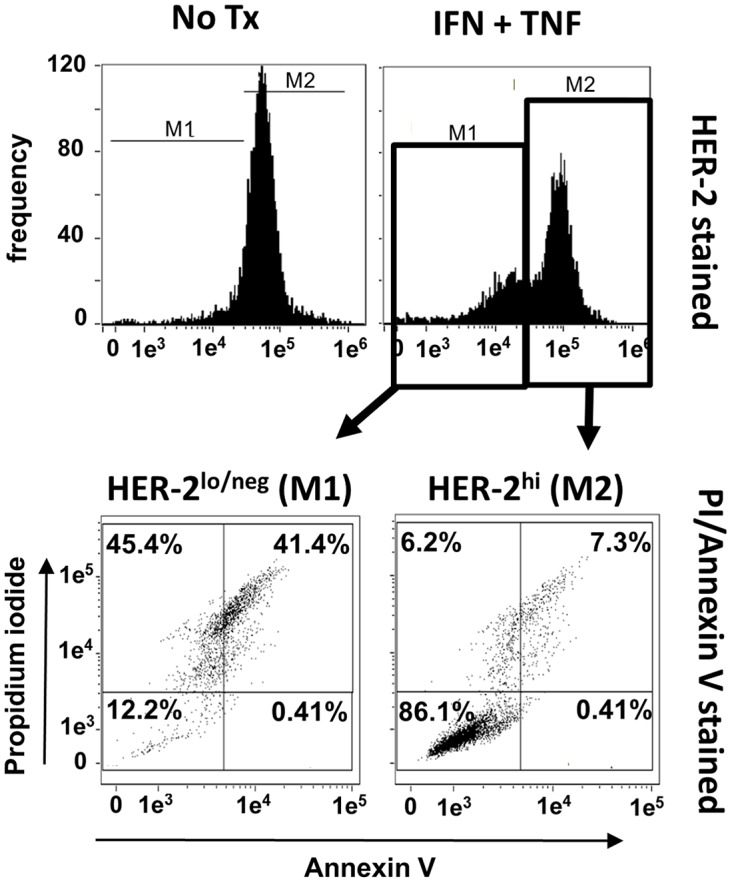
Loss of HER-2 expression is associated with apoptosis. SKBR3 cells were cultured alone or with TNF-α and IFN-γ for 48 hours, harvested, and simultaneously stained with APC-conjugated anti-HER-2 antibody, Annexin V and PI and analyzed via flow cytometry. Upper left; expression of HER-2 in untreated group, upper right; expression of HER-2 in cytokine-treated group. For cytokine-treated group, two gates were defined. M1 gate contained cells with depressed HER-2 expression, M2 contained cells with high retained HER-2 expression. These separate populations were individually analyzed for Annexin V and PI staining (lower panels). Histograms and associated dot plots representative of 3 separate experiments with similar results.

The preceding experiments indicated that paired Th1 cytokines induce both apoptotic cell death and down-regulation of surface HER-2 expression in a variety of murine and human breast cancer lines. We next sought to determine how closely this loss of growth factor receptor was associated with cell death. To accomplish this, we focused on the human SKBR3 cell line. These cells were cultured in the presence and absence of IFN-γ plus TNF-α for 48 hours, harvested, and stained simultaneously with fluorescently-labeled APC-conjugated anti HER-2 antibody, FITC-labeled AnnexinV and propidium iodide (PI), then subjected to multicolor FACS analysis. As before, untreated cells expressed high levels of surface HER-2 protein (Figure 6, upper left panel). For cytokine-treated SKBR3 cells, HER-2 expression is starting to be suppressed at 48 h, but the histogram at this time point reveals bimodality, with a population of cells clearly down-regulating surface HER-2, and another yet retaining high expression. This bimodality allowed us to define logical gates for high HER-2 expression versus low/negative HER-2 expression, and to analyze these populations separately for markers of apoptosis (Figure 6 lower panels). We found that the HER-2^hi^ populations had few AnnexinV^pos^/PI^pos^ cells, with the vast majority in the viable, double-negative quadrant (Figure 6 lower right panel). In contrast, with the HER-2^low/neg^ population, the situation was completely reversed, with high numbers of AnnexinV^pos^/PI^pos^ (i.e. apoptotic) cells, and few remaining viable, double-negative events (Figure 6 lower left panel). This study indicates a close association between down-regulation of HER-2 expression and apoptotic cell death for SKBR3 cells.

### Small molecule agonists of caspase 3 mimic, while its inhibitors block, Th1 cytokine effects

**Figure 7:**
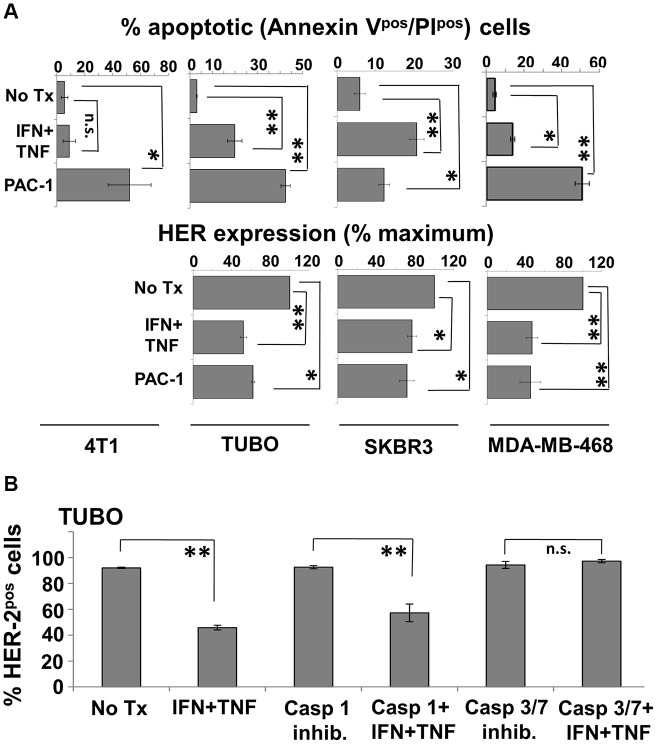
Caspase 3 agonist induces apoptosis and HER-family loss while caspase 3 antagonist prevents cytokine-induced HER-2 loss in breast cancer cell lines. (**A**) Murine 4T1 and TUBO, and humanSKBR3 and MDA-MB-468 cells were cultured alone, with Th1 cytokines, or with caspase-3 agonist PAC-1 (10 μM). Murine lines were harvested 72 hours post-treatment, and human lines 48 hours post-treatment, stained with FITC-Annexin V, PI (upper panels) or with anti-HER antibodies (lower panels), and subjected to flow cytometric analysis. Values are expressed as % total apoptotic cells (AnnexinV^pos^/PI^pos^), and percent maximal HER expression +/− SEM. (**B**) TUBO cells were cultured alone or in the presence of IFN-γ plus TNF-α. To these groups were added either no additional treatment, caspase-1 inhibitor (50 μM), or caspase 3/7 inhibitor I (50 μM). Cells were incubated for 72 hours, harvested, and stained for HER-2 expression and analyzed via flow cytometric analysis. Results are expressed as % HER-2^pos^ cells +/− SEM (^*^ *p* </=.05; ^**^ *p* </=.01; n.s. not significant).

The preceding studies indicated that Th1 cytokine-treated cells lose HER-2 surface expression and die through an apoptotic mechanism, while activating the executioner caspase-3. They do not prove, however, that caspase 3 activity is essential to these processes. We therefore sought to further delineate the role of caspase 3 by testing whether small molecule agonists of this caspase were capable of mediating the same biological effects on breast cancer lines as Th1 cytokines, and whether the effects of cytokines could be blocked by antagonists of caspase 3. We began these studies by examining the effects of PAC-1, a highly selective caspase-3 agonist, on the Th1 cytokine-sensitive murine TUBO cells, and insensitive murine 4T1 cells, as well as sensitive human lines SKBR3 and MDA-MB-468. Cells were cultured in the presence of activator (10 µM), dual Th1 cytokines, or medium alone for 72 hours. Cells were then harvested and stained with Annexin V and propidium iodide to determine apoptotic status, while TUBO and SKBR3 cells were also stained with either anti-rat or anti-human HER-2 antibody, and MDA-MB-468 cells were stained with anti-HER-3 antibody. The 4T1 cells, because of their HER-negativity, did not receive these additional stains. All cell preparations were then subjected to flow cytometry. Analysis indicated that, as expected, 4T1 cells did not undergo apoptosis in response to Th1 cytokines (Figure 7A, upper left panel). They were, however, sensitive to PAC-1; on average half of the cells treated with this agonist became double-positive for Annexin V and PI at this timepoint compared with untreated cells. On the other hand, TUBO, SKBR3 and MDA-MB-468 cells all underwent significant apoptosis in response to both PAC-1 and Th1 cytokines (Figure 7A upper center and right panels). Interestingly, PAC-1 also induced loss of HER-2 expression in TUBO and SKBR3 cells comparable to that caused by Th1 cytokine exposure, and also elicited a similar loss of HER-3 in MDA-MB-468 cells (Figure 7A lower panels), suggesting caspase 3 activation precedes HER loss.

We next turned our attention to caspase antagonists. TUBO cells were treated with Th1 cytokines alone, cytokines plus the caspase 3/7 antagonist (5-[(S)-(+)-2-(Methoxymethyl)pyrrolidino]sulfonylisatin) or as a control, Th1 cytokines plus the Caspase I inhibitor VI (Z-VAD-fmk). After 72 hours, TUBO cells were harvested, stained for surface rHER-2 and subjected to flow cytometry analysis. As expected, we replicated Th1 cytokine-induced loss of rHER-2 expression on TUBO cells (Figure 7B). This loss was not prevented in the presence of the Caspase I inhibitor. However, TUBO cells treated with the Caspase 3/7 inhibitor did not show any evidence of cytokine-induced loss of rHER-2, strongly suggesting a critical role for caspase-3 in the loss of HER-2 surface expression.

## Discussion

At one time, CD8^pos^ CTL were considered to be the most important effector lymphocytes for the control of tumors. However, it is becoming increasingly clear that CD4^pos^ Th cells, particularly those of the IFN-γ^hi^/TNF-α^hi^ Th1 phenotype, play a critical and in some instances a possibly defining role in anti-tumor immunity [16]. In addition to our vaccine studies, our group has recently discovered a number of surprising associations between Th1 immunity and breast cancer. For example, we have demonstrated in healthy donors a surprisingly high degree of pre-existing Th1 immunity against HER-2. Interestingly, this immunity is diminished in patients with early HER-2^pos^ breast cancer (DCIS), and further depressed, sometimes to the point of non-detection, in patients with more advanced invasive HER-2^pos^ tumors [17]. Such losses are not observed in those with HER-2^neg^ breast disease. In follow-on retrospective studies, patients who had invasive HER-2^pos^ breast cancer were treated with neoadjuvant chemotherapy plus trastuzumab [18]. A fraction of these patients achieved pathologic complete responses (pCr) to therapy; i.e. no tumor was detectable after completion of drug therapy. When HER-2 Th1 immunity was compared in the pCR versus non-pCR group, it was found that higher retained Th1 immunity was independently associated with pCR [18]. Another study looked at disease recurrence in HER-2^pos^ invasive breast cancer patients previously treated with chemo plus trastuzumab. As before, higher retained Th1 immunity against HER-2 correlated with the better outcome, in this case longer disease-free survival [19]. Interestingly, when four patients with low anti-HER-2 Th1 immunity who did not experience pCR to chemotherapy plus trastuzumab were vaccinated with HER-2-pulsed IL-12-secreting dendritic cells, their anti-HER-2 immunity levels were restored to the range of healthy individuals [18]. In addition, we showed that Th1 cytokines plus the monoclonal antibody drug Trastuzumab worked cooperatively to sensitize HER-2-expressing tumors to lysis by HER-2-specific CTL *in vitro* [20]. Taken together, these studies indicate a critical role for Th1 immunity in the control of breast cancer, and also suggest the tantalizing possibility that boosting anti-HER-2 Th1 immunity could vastly improve responses to conventional therapy. However, a critical, unaddressed question posed by these studies is, by what possible mechanisms do Th1 cells promote immunity against HER-2^pos^ breast (and perhaps other) cancers?

One way that Th1 responses could participate in tumor control is through a process whose understanding evolved from Burnett’s the original “immunosurveillence” hypothesis [21], and is known as “immunoediting” [12]. The immunoediting hypothesis poses that the adaptive immune system is capable of sculpting tumor phenoypes during the process of oncogenesis, and these influences occur in three phases [22]. The first is “elimination”. As normal cells transform into cancerous ones, changes in gene expression can trigger immune responses capable of destroying all of the altered cells, protecting the body. However, if some malignant cells survive this attack, the “equilibrium” phase is entered. Here, constant immune pressure holds the transformed cells in check, even though it is incapable of destroying them outright. During this phase, the actual “immunoediting” occurs. Malignant cells that acquire, through genetic instability, the characteristics that allow them to resist immune destruction (e.g. antigen loss, acquired resistance to immune effector mechanisms, or acquired immunosuppressive qualities) begin to multiply and break containment. In the final phase, “escape”, the malignant cells have acquired enough changes to allow them to multiply unchecked, and uncontrolled tumor growth results. Thus the immune system is complicit in selecting for the very tumor phenotypes it is incapable of destroying. Interestingly, IFN- γ and lymphocytes are thought to be critical for immunoediting [23, 24, 25, 26], and the cytokine IL-12 has been long known important for driving IFN-γ-secreting Th1 type lymphocytes [27].

In our previously-published clinical trial we used HER-2 peptide-pulsed, IL-12-secreting dendritic cells as vehicles for vaccinating against an early form of HER-2^pos^ breast cancer [8, 9, 10]. This vaccine induced strong, long-lasting Th1 immunity against HER-2, eliminated disease in 18% of subjects and also induced, in about half of the patients, strong loss of HER-2 expression in tumors excised after vaccination. Because our vaccine caused an apparent alteration in tumor phenotype (HER-2^pos^ to HER-2^neg^), under conditions known to be important in classical immunoediting (e.g. polarized type-1 responses), we termed the observed vaccine effect “targeted immunoediting” [10, 28]. We argued that the targeted immunoediting approach was beneficial, since HER-2 expression is associated with invasion and overall poor prognosis [29, 30, 31, 32], and its elimination left behind a residuum of disease with less aggressive characteristics. However, a possible alternate interpretation is that elimination of HER-2 expression is deleterious in the estrogen receptor-negative (ER^neg^) patient subpopulation, because the resulting ER^neg^/HER-2^neg^ phenotype is by definition “triple-negative”, and part of a subset of tumors considered notoriously difficult to treat, since there are currrently fewer targeted treatment options for this phenotype [33].

The data generated in the present studies, however, suggest that we may have previously misidentified at least some portion of our vaccine effects as a form of induced, targeted immunoediting. True immunoediting should produce relatively stable alterations in tumor phenotype (e.g. antigen loss variants). The loss of HER-2 expression, though selective (EpCAM expression was not similarly affected), was shown to be quickly reversed if cytokines were withdrawn prior to full commitment to cell death (Figure 5A), indicating a lack of stability in the HER-2^neg^ cellular phenotype. This observation instead implies that HER-2 expression can be simply regulated by the presence of certain cytokines, and HER-2 loss was somehow tied to the multistep process of programmed cell death. Indeed, we showed that SKBR3 cells analyzed after a 48-hour exposure to Th1 cytokines (a timepoint prior to maximal apoptotic cell death) displayed two distinct cell populations: One with high retained HER-2 expression, and one with diminishing HER-2 expression. For the tested SKBR3 cell line, the HER-2^hi^ population contained few apoptotic cells (7.3%) while the HER-2^lo^ population contained many (41.4%; Figure 6). All things considered, it remains possible that vaccine effects could encompass both targeted immunoediting as well as the demonstrated cytokine-induced HER-2 downregulation phenomena.

But why should the expression of HER-2 or other HER-family members be tied to apoptosis? Cellular outputs of either proliferation or death are determined by input signals coming from growth factor (proliferation) or apoptotic (death) signaling pathways. If there are many growth factor/survival signals and few apoptotic signals, cells live and grow. If there are few growth factor/survival signals and many apoptotic signals, cells undergo programmed cell death. It is therefore perfectly reasonable that a cell entering the decision to undergo apoptosis would down-regulate growth factor receptors to eliminate conflicting signals that would hamper this process. The role of HER-2 in acting in opposition to apoptosis thorugh both intrinsic and extrinsic pathways is well documented and recently reviewed [34]. It is therefore not conceptually surprising that HER family members are down-regulated by apoptosis-inducing Th1 cytokines in both murine and human breast cancer lines. A somewhat unexpected finding, however, was the apparent timing of caspase-3 activation with respect to HER-2 loss. We originally anticipated that caspase-3 activation, being considered a later step in the commitment to cellular apoptosis, would occur after suppression of HER-2. In this scenario, HER-2 loss, perhaps regulated transcriptionally by Th1 cytokine exposure, would rob breast cancer cells of critical growth factor signaling, and hasten them toward apoptosis with eventual activation of caspase-3 as one of the final steps toward commitment to apoptosis. However, our finding that a caspase-3 activator (as a single agent) induced HER-2 down-regulation while a caspase-3 inhibitor prevented Th1 cytokine-induced HER-2 loss offers strong supporting evidence that caspase-3 activation precedes, rather than follows HER-2 down-regulation. Although inhibition of caspase-3 and subsequent prevention of HER-2 loss does not prove caspase-3 acts on HER-2 directly, previous studies by others have demonstrated multiple caspase cleavage sites on the cytoplasmic domain of HER-2, and that HER-2 can be digested by caspase-3 before becomming completely degraded in the proteasome [35]. Despite this, it should also be noted that we detected caspase-3 activation after only a 5 hour exposure to Th1 cytokines, but HER-2 loss does not begin to be detected until 48–7 2 hours after treatment. There may therefore be both direct and indirect roles for caspase-3 in the loss of HER-family surface expression as a consequence of Th1 cytokine exposure. The precise mechanisms of Th1 cytokine-induced changes in breast cancer cells clearly warrants further investigation.

An interesting and somewhat paradoxical finding was that HER-2 and perhaps HER-3 expression is associated with Th1 cytokine sensitivity, even though these RTKs are powerfully down-regulated by these same cytokines. This is evidenced by the fact that of the examined murine tumors, HER-2^pos^ TUBO and MMC15 cells undergo apoptosis in response to Th1 cytokines, while 4T1 cells (which expressed none of the rodent homologs for EGFR, HER-2 or HER-3) were very resistant. In addition, forced overexpression of HER-2 enhanced cytokine susceptibility in human breast lines [17]. The lack of sensitivity of 4T1 cells to Th1 cytokines was not a result of differential expression of cytokine receptors, since we showed via FACS analysis that both susceptible TUBO and resistant 4T1 stained comparably for TNF-α and IFN-γ receptors (Supplementary Figure 1). Despite differences in susceptibility to Th1 cytokines, the caspase-3 agonist PAC-1 induced comparable levels of apoptosis in both TUBO and 4T1 cells. This suggests that some factor farther upstream from this executioner caspase is responsible for the differences in Th1 cytokine susceptibility between these two cell lines. A possible explanation for the differences in susceptibility that accounts for the differential expression of HER-family members entails the concept of oncogene addiction [36]. Oncogene addiction describes a tumor cell’s dependency upon the expression of an oncogene for its survial; eliminating the contribution of the addictive oncogene will result in a dramatic loss of cell viability. If the examined murine and human cell lines are addicted to the expression of the HER-2, then the Th1 cytokine-induced suppression of this oncogene constitutes an insult that cannot be tolerated if the suppression remains constant. In contrast, a cell line that expresses no HER-family proteins by definition cannot be addicted to these oncogenes. So long as other important oncodrivers outside the HER family are not eliminated by Th1 cytokine exposure, such tumor cells would be resistant to cytokine-induced cell death. Another posible explaination would be that expression of some HER family members alters intracellular signaling through some incompletely understood pathway that under the appropriate conditions actually promotes apoptosis. For example, it was recently demonstrated that a proteolytic fragment of HER-2 produced by caspase action can translocate to the mitochondria and initiate apoptosis via the intrinsic pathway [35]. This would explain why expression of HER proteins actually enhanced sensitivity to induced apoptosis.

It should be noted, however, that breast cancer cell lines display considerable heterogeniety in response to Th1 cytokines. Not all HER-expressing lines are equally sensitive to induced apoptosis, and not all lines that undergo apoptosis show strong HER loss. Such heterogeniety is also apparent in our HER-2 dendritic cell vaccine trials for DCIS [8, 9, 10] where 18.5% of subjects showed complete pathological responses (i.e. all observable tumor cells died) while other subjects showed apparent partial reductions in tumor volume, while still others showed no discernable reductions in disease at all. Furthermore, post-vaccine reductions in HER-2 expression in this trial were only seen for about half of the subjects with residual tumor. There are at least two possible explanations for such observations, which are not mutually-exclusive. The first possibility is that for some individuals, not enough Th1cytokines are available at the site of disease to mediate observable changes to the tumors. In our *in vivo* studies we endeavored to use physiologically-plausable cytokine concentrations, but it is difficult to know with certainty the true *in vivo* concentrations at sites of DCIS in vaccinated individuals. In addition, we have shown in other studies that T cells from vaccinated individuals that are stimulated with HER-2 recall peptides produce enough cytokines to induce caspase 3 activation of SKBR3 cells in a transwell assay [17], even when antigen-specific cells constitute less than 1% of the total T cells. This suggests that relatively few cells may be required to affect tumor death at a distance. The second possibility is that the pathways regulating cytokine, HER-2 and apoptotic signaling are somewhat variable from one cell line to another, and also between individual cancers. Although either of these possibilites are reasonable, we acknowledge the limitations of the present *in vitro* studies in determining with certainty the true mechanism of immune-mediated alterations in tumor cells, which may await future animal model studies to positively delineate. Nonetheless, it is apparent that the mechanisms of Th1 cytokine-mediated apoptosis warrants additional investigation as a likely mechanism contributing to the observed vaccine effects. Deliniation of these pathways will allow us to modify and improve our present vaccine therapy, perhaps by the addition of targeted drugs that will enhance cytokine effects by amplifying apoptotic signaling pathways, or further restricting growth factor signaling, thus increasing response rates to therapeutic vaccination.

## Materials and Methods

### Reagents

Cytokines: Recombinant mouse and human IFN-γ and TNF-α were purchased from Peprotech (Rocky Hill, NJ, USA).

Caspase activators, inhibitors and antibodies: Procaspase-activating compound, PAC-1 was purchased from Tocris Bioscience (Avonmouth, Bristol UK), Caspase 3/7 inhibitor ((5-[(S)-(+)-2-(Methoxymethyl)pyrrolidino]sulfonylisatin), Caspase-1 inhibitor VI (Z-YVAD-Fmk), Caspase-1 p10 antibody, Caspase-3 antibody (H-277), Caspase-6 p10 antibody and Caspase-7 p10 antibody were obtained from Santa Cruz Biotechnology (Santa Cruz CA, USA). Actinomycin D was purchased from Sigma (St Louis, MO, USA) and cleaved caspase-3 (Asp175) antibody was purchased from Cell Signaling Technology (Danvers, MA, USA).

Antibodies and cellular stains: anti-rodent HER-2, FITC-labeled anti-mouse Ig and Alamar Blue were purchased from Sigma-Aldrich (St Louis, MO, USA), APC-labeled anti-human HER-2, FITC-AnnexinV and propidium iodide were obtained from BD biosciences (San Jose, CA, USA), APC-labeled Anti-human HER-3 and PE-labeled anti-mouse CD120a (TNF-αR1) were purchased from Biolegend (San Diego, CA, USA), PE-labeled anti-mouse CD119 (IFN-γR1), labeled IgG isotype controls, FITC-labeled anti-human EpCam and APC-labeled anti-mouse EpCam were obtained from eBioscience (San Diego, CA, USA), HRP-conjugated anti-mouse and anti-rabbit IgG were purchased from Santa Cruz Biotechnology (Santa Cruz CA, USA), anti-β-actin was obtained from Millipore (Billerica, MA, USA) and Trypan blue dye was purchased from Lonza Bio Whittaker (Allendale, NJ, USA).

### Cell lines and culture

Murine breast cancer cell lines transgenic for rat ErbB2 (homolog to human HER-2, henceforward referred to as “rHER-2”) TUBO (spontaneously arising from Balb-NeuT mice) and MMC15 (spontaneously arising from FVB-neu-N mice) were kind gifts of Drs. Guido Forni (University of Turin), and Li-Xin Wang (Cleveland Clinic), respectively. Murine 4T1, human HER-2^neg^/HER-3^pos^ MDA-MB-468 and human HER-2^pos^ SKBR3 breast cancer lines were obtained from the American Type Culture Collection (Manassas, VA, USA). 4T1 cells were obtained from the American Type Culture Collection (ATCC, Manassas, VA, USA). The human HER-2^neg^/HER-3^pos^ breast cancer cell line MDA-MB-468 was likewise purchased from ATCC, which authenticates their lines via short tandem repeat profiling. All lines were cultured in RPMI medium supplemented with 10% FBS except SKBR3, which was cultured in McCoy’s medium. All lines were immediately cultured and expanded upon receipt with multiple aliquots re-frozen to establish short-passage stocks. Individual stock cultures were maintained in serial passage for no more than 6 months to minimize drift effects. Cultures were maintained by serial passage in 75 cm^2^ flasks (Corning) at 37°C 5% CO_2_. For individual experiments, cells were seeded in 24-well cluster plates and treated with various combinations of cytokines, caspase agonists and inhibitors. Cells were then harvested and subjected to analysis 24–9 6 hours later.

### Alamar blue assay

Cells seeded into 24-well cluster plates were subjected to various treatments and cultured for 96 hours, after which 20 µl of 0.7 mg/ml stock concentration of Alamar Blue dye was added to each well. After approximately six hours additional incubation, the optical density of each well was read at 630 nm using a BioTek ELx800 spectrophotometer.

### Trypan blue exclusion assay

Viable cell counts were determined by harvesting treated cells at 24, 48, 72 and 96 hours and staining with Trypan Blue dye. Dye-excluding cells were observed microscopically using an Olympus CX2 inverted microscope and enumerated with the aid of a hemocytometer.

### Photomicroscopy

Cells were seeded in 24 well cluster plates, treated with various cytokine combinations or actinomycin D (positive control), and observed each day via phase contrast light microscopy and photographed at 24 and 96 hours using a Olympus CKX41 inverted microscope at total magnification of 100×, using a Hamamatsu camera and Cell Sens software.

### Apoptosis assays

AnnexinV/PI assay staining: Cells were seeded in 24 well cluster plates and treated with cytokines in presence or absence of PAC-1. Cells were then harvested at 24, 48, 72 and 96 hours post-treatment. Harvested cells were washed and resuspended in FACS buffer (PBS + 1% FBS + 0.01% sodium azide), and stained with FITC-AnnexinV (4 µl) and PI (2 µl). Cells were incubated at 4°C for 20 min, washed and subjected to flow cytometry using an Amnis Flow Sight flow cytometer and analyzed by IDEAS analysis suite V6.0. Cells exhibiting AnnexinV^pos^/PI^pos^ phenotype were defined as apoptotic.

### TUNEL assay

Apoptotic cells were detected using Flow TACS^TM^ Apoptosis Detection Kit (Trevigen, Gaithersburg, MD, USA) according to manufacturer’s protocol. Briefly, cells were seeded in 24 well cluster plates and treated with cytokines. Cells were harvested at 72 and 96 hours post-treatment and then incubated in labeling buffer with terminal deoxynucleotidyl transferase (TdT) and biotinylated nucleotides. After washing, cells were incubated with Streptavidin-Fluorescein solution and analyzed by flow cytometry using an Amnis Flow Sight flow cytometer running IDEAS analysis software.

### Detection of surface expression of HER-family proteins

Harvested cells were washed and resuspended in 50 ul FACS buffer (PBS + 1% FBS + 0.01% sodium azide) to prepare them for staining with specific antibodies or their isotype-matched controls. Murine tumor lines were incubated with unconjugated murine anti- rodent HER-2 antibody followed by FITC-conjugated anti-mouse secondary antibody, or stained directly with APC-conjugated anti-EpCAM, PE-conjugated IFN-γR or PE-conjugated TNF-αR1.Human cell lines were stained with APC-conjugated anti HER-2 or anti-HER-3 antibody. Stained cells were incubated at 4°C for 30min, washed and analyzed for HER-2 expression by flow cytometry using an Amnis Flow Sight flow cytometer and IDEAS analysis software.

### Western blot analysis

TUBO and 4T1 cells were seeded in 6-well cluster plates at a density of 5 × 10^4^cells/well in RPMI-1640 media supplemented with 10% FBS and treated with IFN-γ and TNF-α, or no treatment as negative control. Positive control was treated with the pro-apoptotic agent, actinomycin D (10 µM). The plates were then incubated at 37°C for 5 hours. Cells were harvested by trypsinization, washed with ice-cold PBS and resuspended in cold RIPA extraction buffer containing protease and phosphatase inhibitors. The cells were incubated on ice for 30 min and then centrifuged at 14,000 × g for 20 min at 4°C to obtain a clear extract. The total concentration of protein was determined by Bradford assay. Total protein (30 μg) of each lysate was loaded onto 10% polyacrylamide gels (Biorad) and separated by electrophoresis. After electrophoresis, the proteins were electrotransferred onto nitrocellulose membranes, and the membranes were blocked using 1% BSA for one hour. Membranes were then incubated with primary antibodies (cleaved caspase-3 (Asp175), procaspase 1, procaspase 3, procaspase 6 and procaspase 7, and β-actin) at 4°C overnight. Subsequently, the membrane was washed with TBST (0.05% Tween-20 in TBS) and incubated with corresponding anti-mouse or anti-rabbit immunoglobulin G-horseradish peroxidase-conjugated secondary antibody for one hour at room temperature. The membranes were then washed again with TBST. Protein bands were visualized using enhanced chemiluminescence (ECL) detection kit (Pierce) and GE Luminescent image analyzer using Image Quant LAS4000 software. Protein band intensities were analyzed quantitatively with ImageJ.

### Statistical analysis

Quantitative data are presented as means ± SEM. The significance of difference was evaluated with the one-way ANOVA test. A *p* value of </=0.05 was considered statistically significant. Statistical analyses were performed in SPSS version 22 (IBM Corp).

## SUPPLEMENTARY MATERIALS



## References

[1] Ménard S , Fortis S , Castiglioni F , Agresti R , Balsari A . HER2 as a prognostic factor in breast cancer. Oncology. 2001; 61:67–72. 10.1159/000055404. 11694790

[2] Bae SY , La Choi Y , Kim S , Kim M , Kim J , Jung SP , Choi MY , Lee SK , Kil WH , Lee JE , Nam SJ . HER3 status by immunohistochemistry is correlated with poor prognosis in hormone receptor-negative breast cancer patients. Breast Cancer Res Treat. 2013; 139:741–50. 10.1007/s10549-013-2570-6. 23722313

[3] Green AR , Barros FF , Abdel-Fatah TM , Moseley P , Nolan CC , Durham AC , Rakha EA , Chan S , Ellis IO . HER2/HER3 heterodimers and p21 expression are capable of predicting adjuvant trastuzumab response in HER2+ breast cancer. Breast Cancer Res Treat. 2014; 145:33–44. 10.1007/s10549-014-2925-7. 24706169 PMC3984415

[4] Neve RM , Lane HA , Hynes NE . The role of overexpressed HER2 in transformation. Ann Oncol. 2001; 12:S9–13. 10.1093/annonc/12.suppl_1.s9. 11521729

[5] Hayashi N , Iwamoto T , Gonzalez-Angulo AM , Ferrer-Lozano J , Lluch A , Niikura N , Bartholomeusz C , Nakamura S , Hortobagyi GN , Ueno NT . Prognostic impact of phosphorylated HER-2 in HER-2+ primary breast cancer. Oncologist. 2011; 16:956–65. 10.1634/theoncologist.2010-0409. 21712485 PMC3228141

[6] Lee-Hoeflich ST , Crocker L , Yao E , Pham T , Munroe X , Hoeflich KP , Sliwkowski MX , Stern HM . A central role for HER3 in HER2-amplified breast cancer: implications for targeted therapy. Cancer Res. 2008; 68:5878–87. 10.1158/0008-5472.CAN-08-0380. 18632642

[7] Dey N , Williams C , Leyland-Jones B , De P . A critical role for HER3 in HER2-amplified and non-amplified breast cancers: function of a kinase-dead RTK. Am J Transl Res. 2015; 7:733–50. 26064441 PMC4455348

[8] Sharma A , Koldovsky U , Xu S , Mick R , Roses R , Fitzpatrick E , Weinstein S , Nisenbaum H , Levine BL , Fox K , Zhang P , Koski G , Czerniecki BJ . HER-2 pulsed dendritic cell vaccine can eliminate HER-2 expression and impact ductal carcinoma *in situ* . Cancer. 2012; 118:4354–62. 10.1002/cncr.26734. 22252842 PMC3330145

[9] Koski GK , Koldovsky U , Xu S , Mick R , Sharma A , Fitzpatrick E , Weinstein S , Nisenbaum H , Levine BL , Fox K , Zhang P , Czerniecki BJ . A novel dendritic cell-based immunization approach for the induction of durable Th1-polarized anti-HER-2/neu responses in women with early breast cancer. J Immunother. 2012; 35:54–65. 10.1097/CJI.0b013e318235f512. 22130160 PMC3241864

[10] Czerniecki BJ , Koski GK , Koldovsky U , Xu S , Cohen PA , Mick R , Nisenbaum H , Pasha T , Xu M , Fox KR , Weinstein S , Orel SG , Vonderheide R , et al. Targeting HER-2/neu in early breast cancer development using dendritic cells with staged interleukin-12 burst secretion. Cancer Res. 2007; 67:1842–52. 10.1158/0008-5472.CAN-06-4038. 17293384

[11] Dunn GP , Koebel CM , Schreiber RD . Interferons, immunity and cancer immunoediting. Nat Rev Immunol. 2006; 6:836–48. 10.1038/nri1961. 17063185

[12] Dunn GP , Bruce AT , Ikeda H , Old LJ , Schreiber RD . Cancer immunoediting: from immunosurveillance to tumor escape. Nat Immunol. 2002; 3:991–98. 10.1038/ni1102-991. 12407406

[13] Yarden A , Kimchi A . Tumor necrosis factor reduces c-myc expression and cooperates with interferon-gamma in HeLa cells. Science. 1986; 234:1419–21. 10.1126/science.3097823. 3097823

[14] Guan YQ , Li Z , Liu JM . Death signal transduction induced by co-immobilized TNF-α plus IFN-γ and the development of polymeric anti-cancer drugs. Biomaterials. 2010; 31:9074–85. 10.1016/j.biomaterials.2010.08.044. 20832854

[15] Braumüller H , Wieder T , Brenner E , Aßmann S , Hahn M , Alkhaled M , Schilbach K , Essmann F , Kneilling M , Griessinger C , Ranta F , Ullrich S , Mocikat R , et al. T-helper-1-cell cytokines drive cancer into senescence. Nature. 2013; 494:361–65. 10.1038/nature11824. 23376950

[16] Galaine J , Borg C , Godet Y , Adotévi O . Interest of tumor-specific CD4 T helper 1 cells for therapeutic anticancer vaccine. Vaccines (Basel). 2015; 3:490–502. 10.3390/vaccines3030490. 26350591 PMC4586463

[17] Datta J , Rosemblit C , Berk E , Showalter L , Namjoshi P , Mick R , Lee KP , Brod AM , Yang RL , Kelz RR , Fitzpatrick E , Hoyt C , Feldman MD , et al. Progressive loss of anti-HER2 CD4^+^ T-helper type 1 response in breast tumorigenesis and the potential for immune restoration. OncoImmunology. 2015; 4:e1022301. 10.1080/2162402X.2015.1022301. 26451293 PMC4589053

[18] Datta J , Berk E , Xu S , Fitzpatrick E , Rosemblit C , Lowenfeld L , Goodman N , Lewis DA , Zhang PJ , Fisher C , Roses RE , DeMichele A , Czerniecki BJ . Anti-HER2 CD4(+) T-helper type 1 response is a novel immune correlate to pathologic response following neoadjuvant therapy in HER2-positive breast cancer. Breast Cancer Res. 2015; 17:71. 10.1186/s13058-015-0584-1. 25997452 PMC4488128

[19] Datta J , Fracol M , McMillan MT , Berk E , Xu S , Goodman N , Lewis DA , DeMichele A , Czerniecki BJ . Association of depressed anti-HER2 T-helper type 1 response with recurrence in patients with completely treated HER2-positive breast cancer: role for immune monitoring. JAMA Oncol. 2016; 2:242–46. 26719971 10.1001/jamaoncol.2015.5482

[20] Datta J , Xu S , Rosemblit C , Smith JB , Cintolo JA , Powell DJ Jr , Czerniecki BJ . CD4(+) T-helper type 1 cytokines and trastuzumab facilitate CD8(+) T-cell targeting of HER2/neu-expressing cancers. Cancer Immunol Res. 2015; 3:455–63. 10.1158/2326-6066.CIR-14-0208. 25791067 PMC4556111

[21] Burnet FM . The concept of immunological surveillance. Prog Exp Tumor Res. 1970; 13:1–27. 4921480 10.1159/000386035

[22] Dunn GP , Old LJ , Schreiber RD . The three Es of cancer immunoediting. Annu Rev Immunol. 2004; 22:329–60. 10.1146/annurev.immunol.22.012703.104803. 15032581

[23] Kaplan DH , Shankaran V , Dighe AS , Stockert E , Aguet M , Old LJ , Schreiber RD . Demonstration of an interferon gamma-dependent tumor surveillance system in immunocompetent mice. Proc Natl Acad Sci U S A. 1998; 95:7556–61. 10.1073/pnas.95.13.7556. 9636188 PMC22681

[24] Street SE , Cretney E , Smyth MJ . Perforin and interferon-gamma activities independently control tumor initiation, growth, and metastasis. Blood. 2001; 97:192–97. 11133760 10.1182/blood.v97.1.192

[25] Street SE , Trapani JA , MacGregor D , Smyth MJ . Suppression of lymphoma and epithelial malignancies effected by interferon gamma. J Exp Med. 2002; 196:129–34. 10.1084/jem.20020063. 12093877 PMC2194011

[26] Enzler T , Gillessen S , Manis JP , Ferguson D , Fleming J , Alt FW , Mihm M , Dranoff G . Deficiencies of GM-CSF and interferon gamma link inflammation and cancer. J Exp Med. 2003; 197:1213–19. 10.1084/jem.20021258. 12732663 PMC2193978

[27] Hsieh CS , Macatonia SE , Tripp CS , Wolf SF , O’Garra A , Murphy KM . Development of TH1 CD4+ T cells through IL-12 produced by Listeria-induced macrophages. Science. 1993; 260:547–49. 10.1126/science.8097338. 8097338

[28] Koski GK , Cohen PA , Roses RE , Xu S , Czerniecki BJ . Reengineering dendritic cell-based anti-cancer vaccines. Immunol Rev. 2008; 222:256–76. 10.1111/j.1600-065X.2008.00617.x. 18364007

[29] Liao N , Zhang GC , Liu YH , Li XR , Yao M , Xu FP , Li L , Wu YL . HER2-positive status is an independent predictor for coexisting invasion of ductal carcinoma *in situ* of the breast presenting extensive DCIS component. Pathol Res Pract. 2011; 207:1–7. 10.1016/j.prp.2010.08.005. 21095069

[30] Harada S , Mick R , Roses RE , Graves H , Niu H , Sharma A , Schueller JE , Nisenbaum H , Czerniecki BJ , Zhang PJ . The significance of HER-2/neu receptor positivity and immunophenotype in ductal carcinoma *in situ* with early invasive disease. J Surg Oncol. 2011; 104:458–65. 10.1002/jso.21973. 21557226 PMC3168675

[31] Slamon DJ , Clark GM , Wong SG , Levin WJ , Ullrich A , McGuire WL . Human breast cancer: correlation of relapse and survival with amplification of the HER-2/neu oncogene. Science. 1987; 235:177–82. 10.1126/science.3798106. 3798106

[32] Press MF , Bernstein L , Thomas PA , Meisner LF , Zhou JY , Ma Y , Hung G , Robinson RA , Harris C , El-Naggar A , Slamon DJ , Phillips RN , Ross JS , et al. HER-2/neu gene amplification characterized by fluorescence *in situ* hybridization: poor prognosis in node-negative breast carcinomas. J Clin Oncol. 1997; 15:2894–904. 10.1200/JCO.1997.15.8.2894. 9256133

[33] Santana-Davila R , Perez EA . Treatment options for patients with triple-negative breast cancer. J Hematol Oncol. 2010; 3:42. 10.1186/1756-8722-3-42. 20979652 PMC2987865

[34] Carpenter RL , Lo HW . Regulation of Apoptosis by HER2 in Breast Cancer. J Carcinog Mutagen. 2013; 2013. 10.4172/2157-2518.S7-003. 27088047 PMC4830426

[35] Strohecker AM , Yehiely F , Chen F , Cryns VL . Caspase cleavage of HER-2 releases a Bad-like cell death effector. J Biol Chem. 2008; 283:18269–82. 10.1074/jbc.M802156200. 18420586 PMC2440621

[36] Weinstein IB . Disorders in cell circuitry during multistage carcinogenesis: the role of homeostasis. Carcinogenesis. 2000; 21:857–64. 10.1093/carcin/21.5.857. 10783304

