# Supplementary material for: T-helper 1-type cytokines induce apoptosis and loss of HER-family oncodriver expression in murine and human breast cancer cells
Source: Oncotarget. 2019 Oct 15;10(57):6006–20. doi: 10.18632/oncotarget.10298 (PMC6800266; doi:10.18632/oncotarget.10298)
Supplement: Supplementary file 1 [file oncotarget-10-6006-s001.pdf]

T-helper 1-type cytokines induce apoptosis and loss of HER-family oncodriver expression in murine and human breast cancer cells

SUPPLEMENTARY MATERIALS

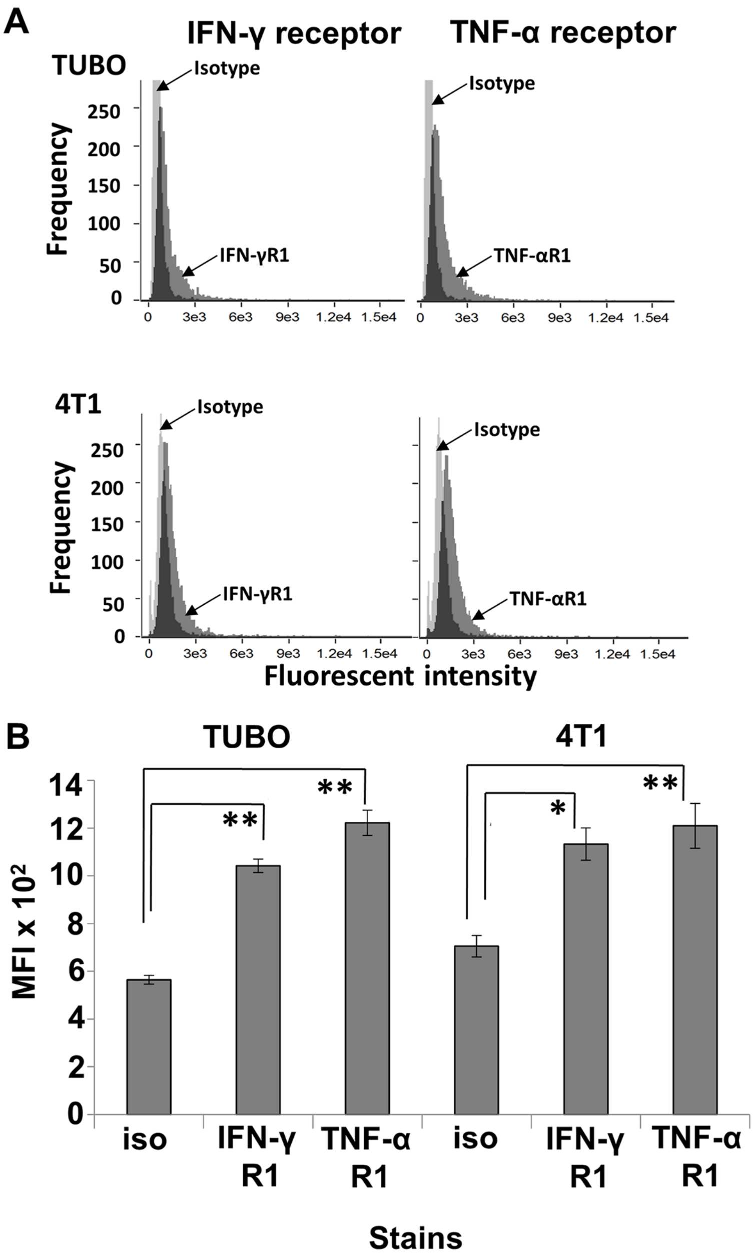

Supplementary Figure 1: Cytokine-sensitive and insensitive cell lines express comparable levels of Th1 cytokine receptors. Untreated TUBO and 4T1 cells were harvested and stained with PE Anti-mouse CD120a (TNF-α R1), PE Anti-mouse CD119 (IFN-γR1) or PE –labeled control IgG and analyzed by flow cytometry. (**A**) Representative histograms of stained TUBO and 4T1 cells. (**B**) Summary analysis of fluorescent values denoted by mean fluorescent intensity (MFI) of PE channel ($^{*}p$ </ = .05; $^{**}p$ </ = .01). Composite results from 3 independent experiments. +/−SEM.

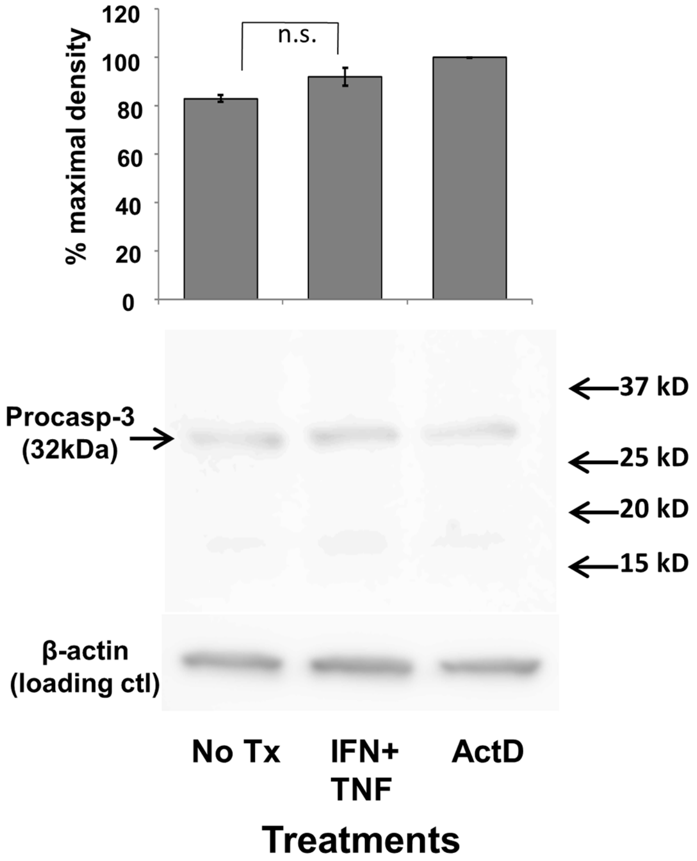

Supplementary Figure 2: Th1 cytokines fail to induce activation of caspase-3 in cytokine-insensitive 4T1 cells. Murine 4T1 cells were cultured alone, in the presence of TNF-α plus IFN-γ, or with actinomycin D for 5 hours, harvested, and subjected to Western blot analysis using anti-procaspase 3 antibodies. Upper panel represents combined data from 3 separate experiments with caspase staining intensity values normalized against β-actin +/- SEM. Lower panel features a representative blot from one of these experiments.

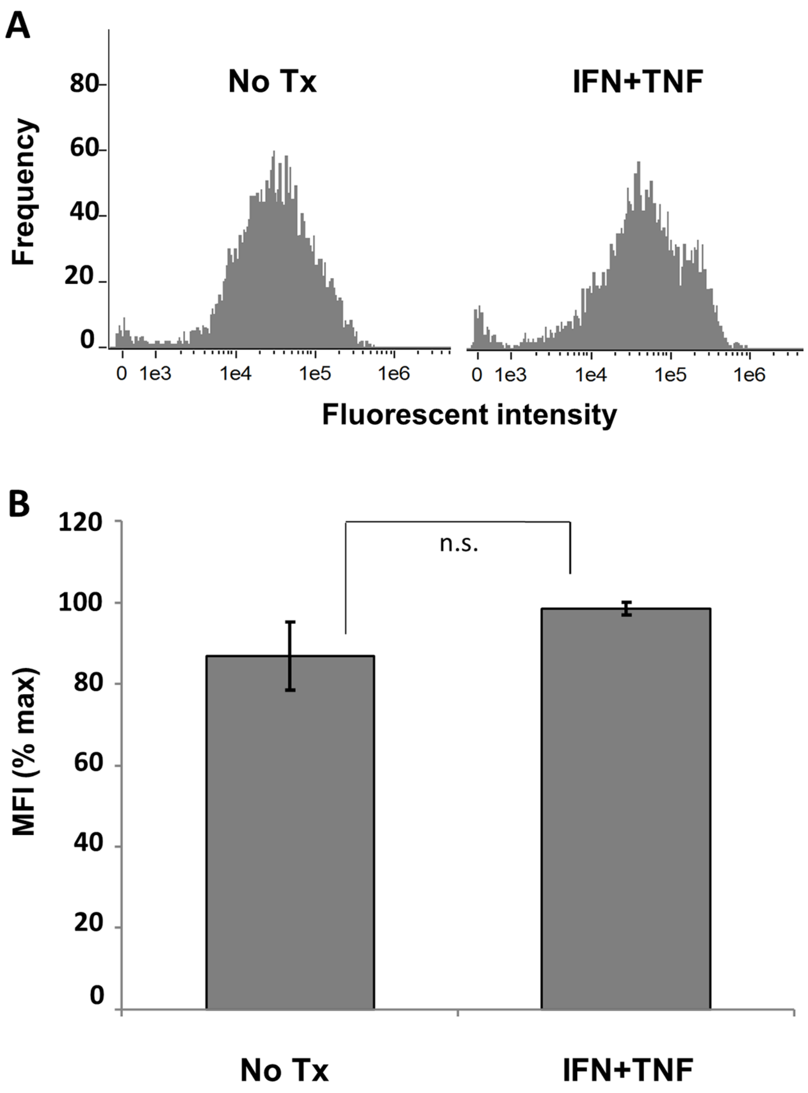

Supplementary Figure 3: Surface expression of EpCAM is unaffected by Th1 cytokines. TUBO cells were cultured alone or in the presence of TNF-α and IFN-γ for 72 hours, harvested and analyzed for EpCAM expression via flow cytometry. (**A**) Representative histograms of untreated and treated TUBO cells. (**B**) Composite data of 3 separate trials. Values represent percent maximal mean fluorescence intensity +/− SEM from 3 separate experiments.
